# Rectus Sheath Hematoma as a Complication in Patients With COVID-19: Clinical and Imaging Findings

**DOI:** 10.7759/cureus.38943

**Published:** 2023-05-12

**Authors:** Emre Emekli, Mesut Yıldırım, Mustafa H Türkkanı, Emel Ödemiş Başpınar

**Affiliations:** 1 Radiology, Etimesgut Şehit Sait Ertürk State Hospital, Ankara, TUR; 2 Chest Diseases, Etimesgut Şehit Sait Ertürk State Hospital, Ankara, TUR; 3 Infectious Diseases and Clinical Microbiology, Etimesgut Şehit Sait Ertürk State Hospital, Ankara, TUR

**Keywords:** hemorrhage, ultrasound, anticoagulation, hematoma, covid-19, rectus sheat hematoma

## Abstract

Objectives

This study aims to investigate the frequency of rectus sheath hematoma (RSH), clinical findings, imaging findings, and prognosis in patients admitted to the hospital due to COVID-19.

Methods

In this retrospective study, the patient's demographic characteristics, known diseases, laboratory values, RSH-related symptoms, the treatment they received, imaging modality used to diagnose RSH, and side and size of RSH were recorded. In addition, the inpatient ward to which the patients were admitted, length of hospital stay, time from the beginning of anticoagulant use to the diagnosis of RSH, and prognosis were noted.

Results

A total of 9,876 patients were admitted to the hospital due to COVID-19 and started anticoagulant treatment. Of these patients, 12 (0.12%) were determined to have RSH (female/male ratio: 5). The prothrombin time, activated partial thromboplastin time, international normalized ratio, hemoglobin, and hematocrit values ​​of 11 patients were within the reference ranges. The mean length of hospital stay was 12 (4.25-22.5) days, and the duration of anticoagulant use was 5.5 (4-10.75) days. RSH was diagnosed using USG in 10 patients and CT in two patients.

Conclusion

There has been an increase in the use of anticoagulants due to COVID-19, and accordingly, RSH is now more frequently diagnosed and has a more fatal course. Female gender, advanced age, severe COVID-19 disease, and elevated d-dimer at the time of presentation can be considered risk factors for the development of RSH. All physicians who treat and follow up on patients with COVID-19 should consider the possibility of RSH in the differential diagnosis of patients with acute abdominal pain and palpable masses. USG should be undertaken as the first-line imaging modality for the diagnosis of patients, but CT may also be necessary to detect RSH in some cases.

## Introduction

Rectus sheath hematoma (RSH) is a rare cause of acute abdominal pain. In the literature, it has been reported that the underlying pathophysiological mechanism of this entity is a rupture in the epigastric arteries or rectus muscles. Furthermore, the use of anticoagulants has been shown to be one of the most important factors causing RSH. RSH has been reported to be responsible for unexplained abdominal pain in approximately 2% of hospitalized patients in the general population [1,2]. In addition to anticoagulant treatment, cough, straining, exercise, hypertension, obesity, previous abdominal surgery, subcutaneous injection, and trauma are known to be predisposing factors for RSH [3]. Today, it is known that in addition to pulmonary involvement, systemic involvement also plays an important role in mortality and morbidity in COVID-19 infection [4,5]. Thromboinflammatory events have been shown to be involved in the mechanism of this disease. The virus activates the infection-related coagulation pathway by attaching to the receptors on the endothelial surface [6]. Accordingly, venous thromboembolism, acute stroke, myocardial infarction, acute venous thromboembolism, and acute venous and arterial thrombotic complications related to COVID-19 have been described in the literature [7-9]. Since thrombotic events have been reported by many studies, it is recommended to initiate prophylactic and anticoagulant treatment in the early phase of COVID-19 disease. Although there is no consensus in the guidelines concerning the dose of treatment, antithrombotic use is currently accepted as a routine treatment [10]. However, although the routine use of anticoagulant therapy is beneficial in the treatment of COVID-19, RSH may develop secondary to this treatment and can follow a fatal course in some cases [1,11]. Therefore, it is important to diagnose RSH in patients with COVID-19 and effectively manage this disease. Due to the relatively lower incidence of RSH among the other causes of acute abdominal pain, all physicians providing care for patients diagnosed with or recovered from COVID-19 should be familiar with this entity to prevent unnecessary laparotomies and even death. In the literature, there are only a limited number of studies on RSH in patients with COVID-19. Therefore, this study aimed to investigate the frequency of RSH, clinical and imaging findings, and prognosis in patients admitted to the hospital due to COVID-19.

## Materials and methods

This research was planned as a retrospective observational study. Ethical approval was obtained for this study from the Ethics Committee of Yıldırım Beyazıt University, Yenimahalle Training and Research Hospital. For the study, patients who were hospitalized due to COVID-19 between March 2020 and July 2022 were identified from hospital records. Among these patients, those with a diagnosis of RSH were noted. During the study period, the hospital was commissioned by the health authority to provide outpatient, inpatient, and intensive care services only for patients with COVID-19. The patients’ demographic characteristics of patients (age, gender), known diseases, laboratory values, symptoms associated with RSH, the treatment they received, imaging modality used to diagnose RSH, and side and size of RSH were recorded. International normalized ratio (INR), activated partial thromboplastin time (aPTT), prothrombin time (PT), d-dimer, hemoglobin (Hgb), hematocrit (Hct), C-reactive protein (CRP), and ferritin were recorded from laboratory data. Furthermore, the hospital unit to which the patients were admitted (inpatient ward, intensive care unit), length of hospital stay, time from the beginning of anticoagulant use to the diagnosis of RSH, and prognosis were noted for all the patients. The severity of COVID-19 was evaluated according to the World Health Organization classification [12]. SPSS Statistics version 22.0 (IBM Corp. Released 2013. IBM SPSS Statistics for Windows, Version 22.0. Armonk, NY: IBM Corp.) was used for statistical analyses. Descriptive statistics were presented as mean ± standard deviation values for continuous data and median, percentage, and range values ​​for discrete data.

## Results

During the study period, a total of 9,876 patients, 4,660 women and 5,216 men, were admitted to the inpatient wards and intensive care unit due to COVID-19, and anticoagulant treatment at therapeutic doses was started in these patients. Of these patients, 12 (0.12%) were determined to have RSH. Ten of these patients (83.3%) were female and two (16.7%) were male (female/male ratio: 5). The mean age of the whole cohort was 54.98 ± 16.71 years, 56.57 ± 17 years for the women and 53.56 ± 16.32 years for the men. The mean age of the patients with RSH was 68.25 ± 19.41 years, and that of the male and female patients with RSH was 59.5 ± 28.99 years and 70 ± 18.63 years, respectively. The demographic and clinical characteristics of the patients are summarized in Table 1.

**Table 1:** Demographic and clinical characteristics of the patients RHS: rectus sheath hematoma; LMWH: low-molecular-weight heparin; UFH: unfractionated heparin; ASA: acetylsalicylic acid; PRBC: packed red blood cell; FFP: fresh frozen plasma; ICU: intensive care unit; USG: ultrasonography; CT: computed tomography; NA: not applicable

Patient number	Age	Sex	Chronic anticoagulant therapy	Anticoagulant treatment	Day of LMWH	Length of ward stay	Length of ICU stay	Modality	RHS side	RHS size (mm)	Management	Mortality
1	36	F	None	LMWH	4	5	NA	US	Right	17x9	Conservative	Alive
2	94	F	None	LMWH	11	28	15	US + CT	Bilateral	59x30	PRBC	Alive
3	65	F	None	LMWH	9	14	NA	US + CT	Right	100x55	Conservative	Alive
4	43	F	None	LMWH	11	12	7	US	Left	100x68	PRBC	Alive
5	68	F	ASA + clopidogrel	LMWH	3	3	NA	US	Left	36x28	Conservative	Alive
6	80	M	None	LMWH	6	15	NA	US + CT	Right	60x39	PRBC	Alive
7	88	F	None	UFH	13	25	25	US	Right	61x28	PRBC	Dead
8	72	F	ASA	LMWH	5	10	10	US	Right	120x38	PRBC + FFP	Dead
9	79	F	None	LMWH	4	12	12	US	Right	80x50	PRBC + FFP	Dead
10	85	F	None	LMWH	10	29	NA	US + CT	Left	54x24	Conservative	Alive
11	40	M	ASA	LMWH	5	4	NA	CT	Left	57x40	Conservative	Alive
12	70	F	ASA	LMWH	2	2	NA	CT	Bilateral	58x34	Conservative	Alive

The mean laboratory values of the patients at the time of presentations were measured as follows: PT, 13.05 (12.5-14.13) sec; aPTT, 23.65 (22.43-26.35) sec; INR, 1.1 (1.03-1.18); d-dimer, 1.3 (0.8-3.5) mg/L; Hgb, 12.35 (11.75-14.25) g/dl; Hct, 38.25% (36.15-43.35%), and CRP, 31.4 (13.73-113.4) mg/dL. In 11 patients, the PT, aPTT, INR, Hgb, and Hct values were within the reference ranges at presentation. Data on ferritin ​​were available for nine patients, for whom the mean ferritin value was calculated as 120 (71.8-503.41) ng/mL. The mean CRP, ferritin, and d-dimer values ​​were above the reference ranges at the time of presentation. Table 2 summarizes the lowest or highest laboratory values ​​of the patients at presentation.

**Table 2:** Laboratory values of the patients Hgb: hemoglobin; Hct: hematocrit; min: minimum; aPTT: activated partial thromboplastin time; max: maximum; INR: international normalized ratio; PT: prothrombin time; CRP: C-reactive protein

Patient number	Admission Hgb/min Hgb (11-17 g/dl)	Admission Hct/min Hct (36-51%)	Admission aPTT/max aPTT (21.6-28.7 sec)	Admission INR/max INR (0.8-1.2)	Admission PT/max PT (10-15 sec)	Admission d-dimer/max d-dimer (0-0.55 mg/L)	CRP (0-5 mg/dL)	Ferritin (4.63-204 ng/mL)
1	11.9/11	36.3/36.1	24.1/28.3	1/1	12.5/12	0.39/0.41	11.2	29.76
2	9.6/4.9	31/15.3	21.8/26.1	1/1.3	11.8/16	4.01/9.17	18.9	76.6
3	12.2/8.2	37.3/25.2	23.1/23.1	1.1/1.1	12.7/12.8	1.04/3.85	164.2	561.93
4	15.9/8.2	50/26.4	23.9/25.1	1.1/1.3	13.6/15.5	26.59/26.59	112.8	112.5
5	12.2/9.8	37.9/29.5	23.2/28	1.1/1.2	13/14.5	1.2/2.65	12	NA
6	14.3/7.5	43.6/23	21.6/24.3	1/1.1	12.2/13	0.72/0.8	113.6	120
7	12.9/9.9	38.6/31.2	26.8/26.8	1.2/1.2	14.2/14.2	26.58/28.73	34.8	541.81
8	11.7/4.9	36.1/16.2	27.5/28	1.2/1.3	14.9/15	1.95/2.65	20.9	67
9	12.5/6.6	39.6/19.9	23.4/25.6	1.1/1.2	13.9/14.2	1.39/10.51	27.9	465
10	10.1/8.2	31.4/25.9	25/25.5	1.1/1.1	13.1/13.5	1.51/1.64	186.5	202.4
11	17/16.4	51.5/49.7	27.7/35.8	2/2.3	22.4/25.5	0.19/0.2	9.7	NA
12	14.1/9.4	42.6/29.5	22.2/23	1.1/1.2	12.5/13	1.1/1.42	110	NA

**Figure 1:**
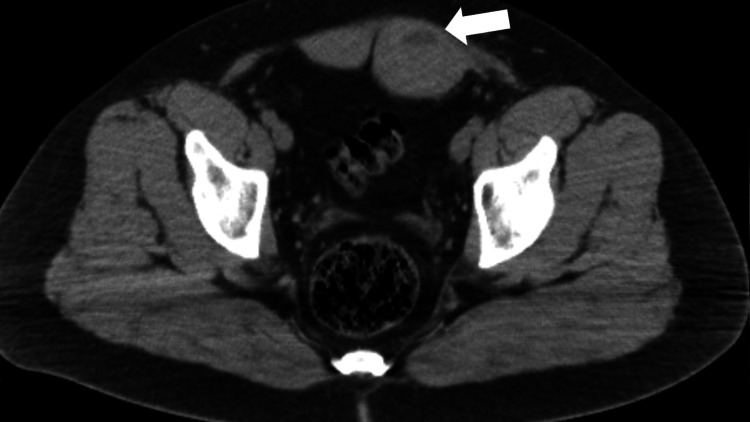
RSH located in the left rectus muscle. Axial CT images (Patient 11)

**Figure 2:**
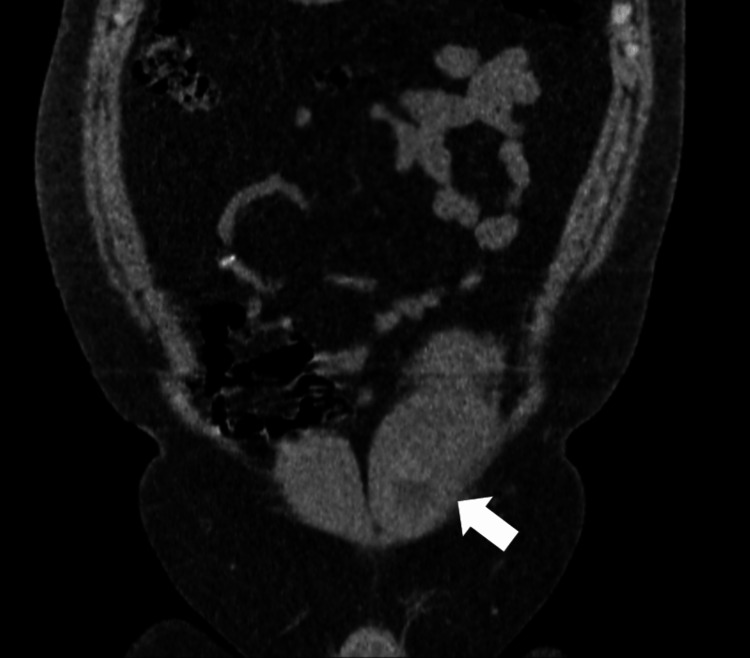
RSH located in the left rectus muscle. Coronal CT image (Patient 11)

According to the COVID-19 disease classification of the World Health Organization, three (25%) patients had critical disease, five (41.7%) had severe disease, and four (33.3%) had moderate disease. At the time of the first hospitalization, nine patients were admitted to the inpatient wards and three to the intensive care unit. Later, one patient was transferred to intensive care due to RSH and another due to the progression of COVID-19. The mean length of hospital stay was 12 (4.25-22.5) days, and the mean duration of anticoagulant use was 5.5 (4-10.75) days. The treatment of all hospitalized patients was arranged following the Guidelines of the Turkish Ministry of Health [13]. Accordingly, Prednol was given to all 12 patients with RSH and low-molecular-weight heparin (LMWH) to 11 of these patients. The doses of LMWH were adjusted during daily visits according to the d-dimer levels and disease severity of the patients according to the same guidelines. All the patients received a therapeutic dose of anticoagulant therapy until the diagnosis of RSH. Nine patients had at least one chronic disease (hypertension in eight, diabetes mellitus in three, coronary artery disease in three, chronic kidney disease in one, asthma in one, and a history of cerebrovascular events in one). Four patients were routinely receiving anticoagulant therapy, which was continued during the hospital stay of the patients until they were diagnosed with RSH. Symptoms before the diagnosis of RSH were abdominal pain in six patients, sudden decrease in Hgb in two patients, low back pain in one patient, general condition disorder and diffuse abdominal pain in one patient, palpable mass in one patient, and ecchymosis in one patient. Six patients were followed up with conservative treatment. Packed red blood cell replacement was performed in the remaining six patients, and fresh frozen plasma replacement was additionally undertaken in two patients. Mortality occurred in three critically ill patients who had been admitted to the intensive care unit. Two of the deceased patients experienced a dramatic drop in Hgb, while the other had widespread abdominal pain and a general deterioration in health status. Therefore, the cause of death in the two cases was determined to be RSH. In the third patient with COVID-19-related poor health, RSH was added to this. RSH was considered a potential factor in the death of this patient. RSH was diagnosed using USG in 10 patients and intravenous contrast-enhanced CT in two. Direct non-contrast CT was performed to determine the etiology in two patients with abdominal pain (Figures 1, 2).

**Figure 3:**
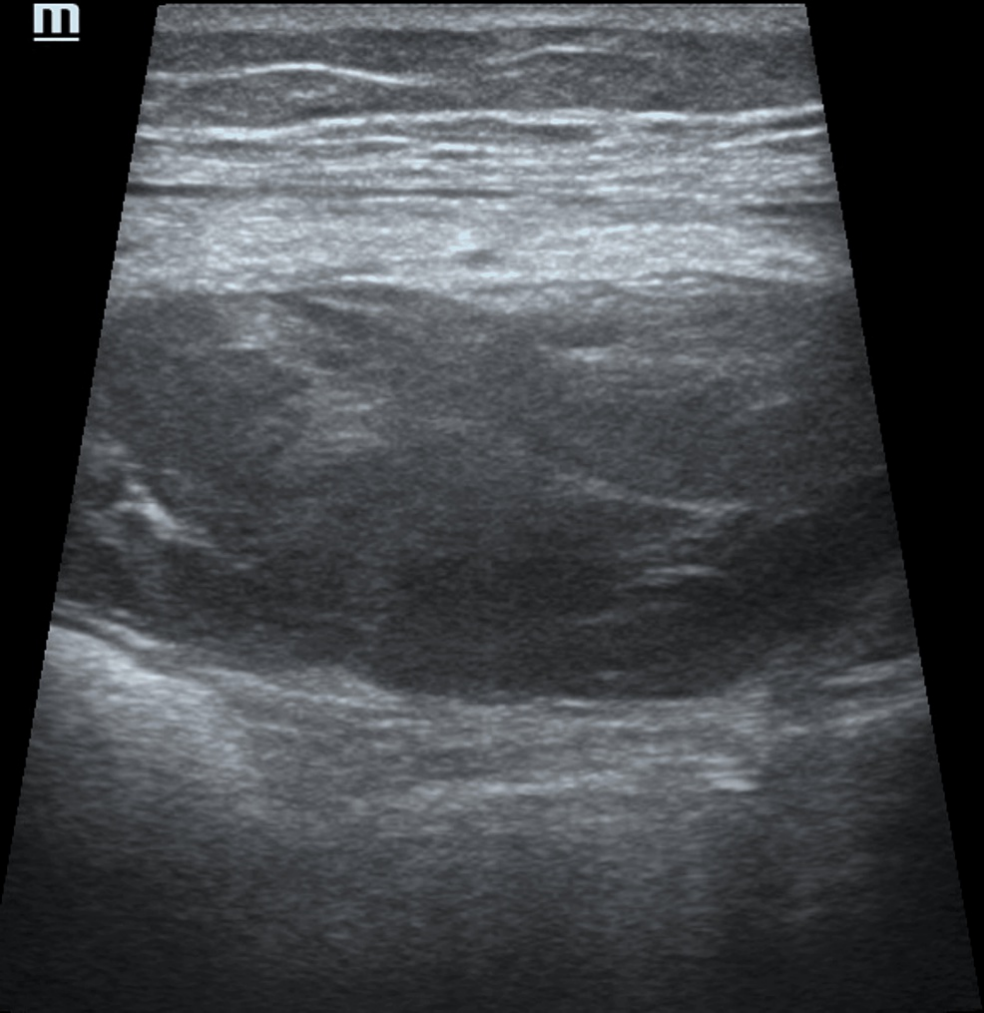
USG image taken in the RSH acute-subacute period with a high-frequency linear probe at the time of first diagnosis (Patient 10)

**Figure 4:**
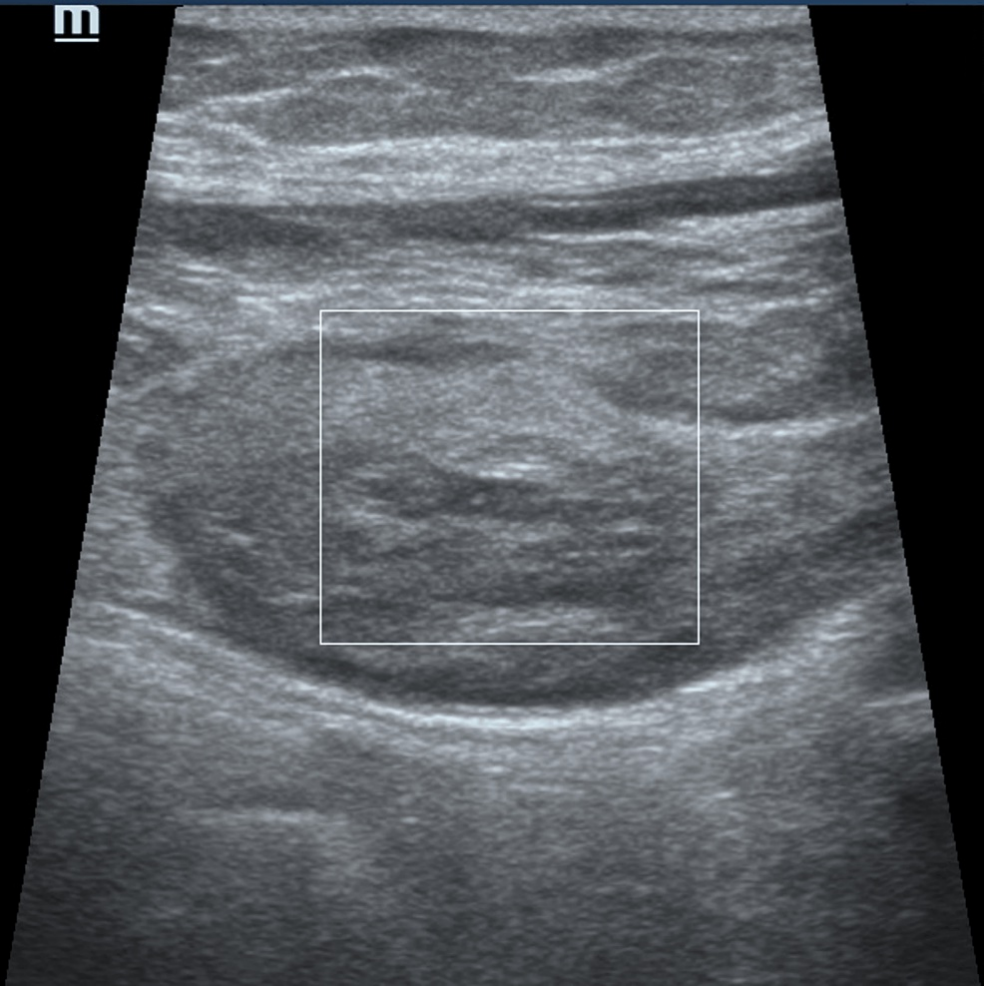
Doppler USG image showing no vascularity region of interest in the lesion (Patient 10)

**Figure 5:**
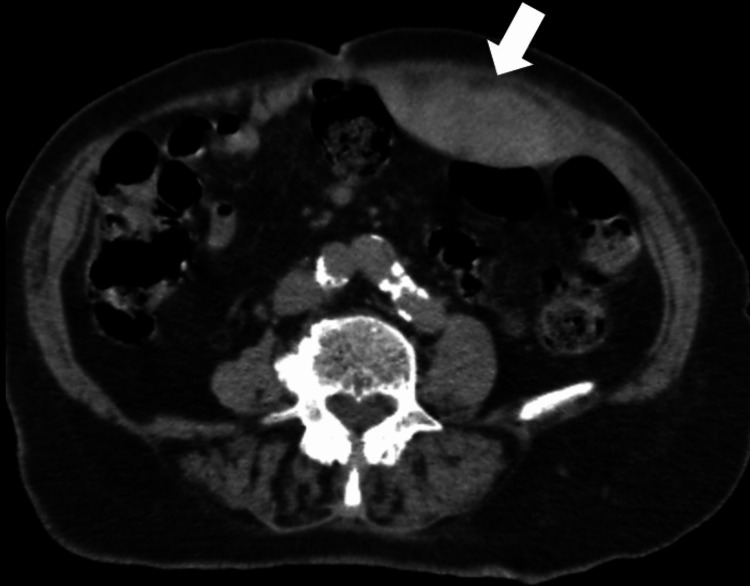
Left RSH (arrow) on axial CT image taken after USG on the same day (Patient 10)

**Figure 6:**
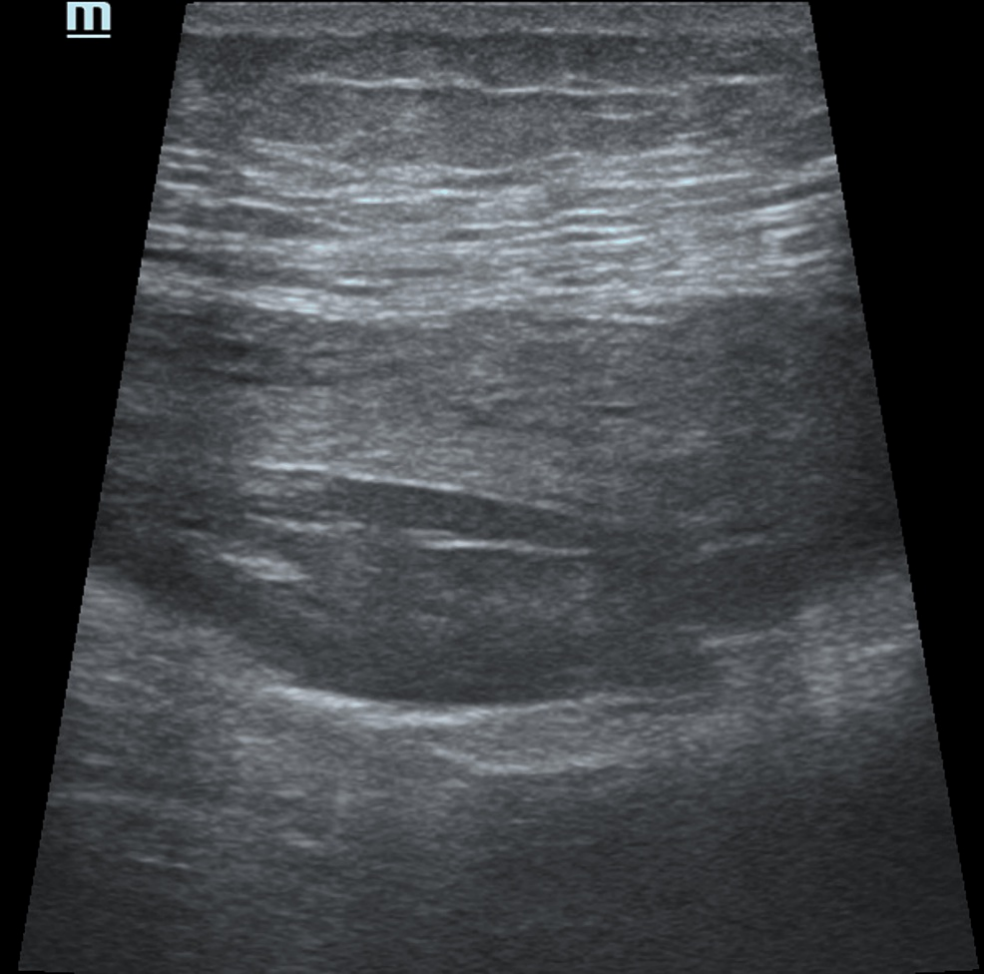
USG image taken four days later after diagnosis showing increased echogenicity of RSH (Patient 10)

In four cases diagnosed with RSH using USG, the diagnosis was subsequently confirmed by CT (Figures 3-6). However, a CT examination could not be performed on three patients who had been directly admitted to the intensive care unit and one patient who had been transferred to the intensive care unit due to the deterioration in her clinical condition. These four patients were followed up with bedside USG. In the remaining two patients, CT was not considered necessary due to their good general conditions and lower decrease in Hgb.

## Discussion

In the literature, RSH is generally described as a rare self-limiting condition. However, it has also been reported to have a fatal course, albeit rarely [14]. It is known that COVID-19 predisposes patients to thrombosis. A previous study reported the rate of arteriovenous thromboembolism as 6.6%, pulmonary thromboembolism as 2.4-35%, isolated DVT of the lower limbs as 0.9-54%, proximal DVT as 0.5-25%, and major bleeding as 2.7% in patients with COVID-19 [9]. Therefore, although there are different anticoagulant recommendations in different guidelines, anticoagulant therapy has been and is still widely used in patients with COVID-19 [8]. Although the mean mortality rate in patients with RSH is reported to be 4%, there are studies showing that this rate reaches 25% with the use of anticoagulants [15]. In a study conducted with COVID-19 cases, RSH mortality was found to be 55.5% [1]. In the present study, the mortality rate was 25%, which is higher than reported for the general population. In a previous study, it was determined that the bleeding rate was 5.6% in critically ill COVID-19 cases receiving anticoagulant prophylactic treatment [16]. In addition, in a study evaluating 5,484 patients hospitalized due to COVID-19, hemorrhage or hematoma was detected in 56 (1%) patients. It was noted that hematoma was detected in 11 of these patients, of whom two (0.04%) had RSH [17]. In another recent study, it was shown that the probability of RSH in patients referred for consultation due to acute abdominal pain was three times higher than reported for the general population [18]. In the current study, we detected RSH in 12 patients (0.12%) among the 9,876 COVID-19 cases. All these data support the higher frequency of RSH in patients with COVID-19 compared to the general population. The study showed that the female-male ratio of RSH was found to be 5:1. Previous studies have reported that RSH is more common in women than in men with a ratio of 2.3:1 due to women having less muscle mass and pregnancy [19]. This ratio was reported to be 1.6:1 in a study conducted with patients with COVID-19 [18]. Consistent with the literature, the majority of patients with RSH were women. It is known that the frequency of RSH diagnosis increases with age. In studies conducted in the general population, the mean age was reported to be in the range of 46-69 years [20]. In two studies investigating the presence of bleeding and hematoma in patients with COVID-19, this rate was determined as 65 years and 78 years, respectively [1,19]. In the present study, nine patients with RSH were aged over 65 years, and the mean age of all the patients with RSH was 68.25 ± 19.41 years. When the laboratory data of the patients were evaluated, there was a difference of 4.35 (2.03-6.58) g/dl and 12.6% (5.98-19.85%) between the presentation and minimum values of Hgb and Hct, respectively. As reported in the literature, Hct values may not always change in small hematomas. However, as in all hemorrhage cases, frequent Hgb and Hct follow-up is recommended to evaluate the need for transfusion or other treatment options [21]. The study determined the mean d-dimer value of the patients as 1.3 (0.8-3.5) mg/L at the time of presentation. In a study conducted with COVID-19 cases that developed hematomas, it was reported that the d-dimer values were elevated in both the mortality and non-mortality groups and higher in the mortality group [17]. In the present study, RSH was detected at the earliest two days after the patients were diagnosed with COVID-19 and started to use anticoagulants. The mean length of hospital stay was 12 (4.25-22.5) days, ranging from 2 to 29 days. In a study conducted on COVID-19 cases, a patient was reported to have bleeding on the fourth day of treatment [17]. Therefore, the study considers that there is no relationship between the duration of treatment and bleeding in patients with COVID-19. Although abdominal pain is the most common symptom described in the literature in patients with RSH, there is no specific symptom that directs the clinician to the diagnosis of RSH [19,20,22]. Therefore, the diagnosis of RSH is mostly made using imaging methods. In the study, 10 patients were diagnosed with RSH using USG, and then the diagnosis was confirmed by CT if needed. The remaining two patients were directly diagnosed with CT. When investigating the etiology of acute abdominal pain, USG is the first-choice method for diagnosis due to its inexpensive and accessible nature and reliable findings [23]. USG is also the method of first choice in the diagnosis and follow-up of RSH [24]. In addition, as in this study, patients with critical conditions can be followed up with bedside USG. Generally, high-frequency probes are used in USG to evaluate muscle injuries and hematomas in the abdominal wall. However, in the presence of fluid collection and large hematomas, convex probes can also be used for diagnosis [25,26]. Imaging characteristics vary according to the time of bleeding. A hematoma is usually visualized as a collection of hypoechoic fluid within 24-48 hours of development, while echogenic solid components begin to increase in the periphery over the hypoechoic center within 48-72 hours. At a more advanced stage, scar tissue begins to form with fibroblast and connective tissue accumulation, and a mass appearance is prominent [26,27]. However, it should be kept in mind that the sensitivity of USG is 80-90% at RSH detection, and it may give false information concerning the origin of the detected mass [24]. The gold standard method for the diagnosis of RSH is a CT examination, allowing for a more definitive diagnosis than USG. The sensitivity and specificity of CT can reach 100% for RSH. It provides more accurate information about the location, size, and extent of the lesion and the nature of the hematoma. It can also be used to exclude other acute abdominal pathologies [2,28]. On non-contrast CT, RSH can typically be visualized as a hyperdense mass (60-80 HU). It is also detected as an isodense or hyperdense mass with a mixed pattern and fluid-fluid level [29,30]. In addition, CT angiography can show whether there is active bleeding into the hematoma in patients with clinical worsening. Bleeding is observed as a high-density area similar to the vessel within the hematoma [30]. In MRI, there are different imaging characteristics in sequences according to the bleeding time, and the diagnosis can be easily made based on these differences. However, MRI has no place in the diagnosis of RSH among routine diagnostic tools, and it is only indicated in cases where the differential diagnosis of a mass is uncertain with CT after five days of hematoma development [20]. The study has certain limitations. First, it was designed retrospectively. Second, the number of patients was small because the diagnosis of RSH is relatively rare. Studies with larger patient populations can further contribute to the literature. Furthermore, only three patients died in our study, and, therefore, the study was not able to compare the mortality and non-mortality groups in terms of risk factors. Such a comparison may provide more useful data for a better understanding of risk factors for RSH-related mortality in patients with COVID-19.

## Conclusions

RSH is generally known as a self-limiting and rarely fatal disease. However, there has been an increase in the use of anticoagulant drugs in both prophylactic and therapeutic doses with the COVID-19 pandemic. Both due to the nature of COVID-19 and secondary to related treatment, bleeding complications increase in these patients. Therefore, RSH has become more common and more fatal. In addition, female gender, advanced age, severe COVID-19 disease, and elevated d-dimer elevation at the time of presentation to the hospital can be considered risk factors for the development of RSH. All physicians who treat and follow up on patients with COVID-19 should consider the possibility of RSH in differential diagnosis, especially in cases with risk factors and acute abdominal pain or palpable abdominal masses. Furthermore, it should be known that USG is the first-choice imaging modality to be performed in the diagnosis of RSH, but CT may also be necessary to diagnose RSH in some cases.

## References

[1] Mahmoudabadi HZ, Hadadi A, Fattahi MR, Kafan S, Ashouri M, Allahbeigi R, Hajebi R (2022). Rectus sheath hematoma in COVID-19 patients as a mortal complication: a retrospective report. Int J Clin Pract.

[2] Abdelmohsen MA, Alkandari BM, Abdel Razek AA, Tobar AM, Gupta VK, Elsebaie N (2021). Abdominal computed tomography angiography and venography in evaluation of hemorrhagic and thrombotic lesions in hospitalized COVID-19 patients. Clin Imaging.

[3] Ortega-Carnicer J, Ceres F (2003). Rectus sheath haematoma with severe haemodynamic compromise after enoxaparin use for unstable angina. Resuscitation.

[4] Mirsadraee S, Gorog DA, Mahon CF (2021). Prevalence of thrombotic complications in ICU-treated patients with coronavirus disease 2019 detected with systematic CT scanning. Crit Care Med.

[5] Okuyucu M, Ozturk O, Atay MH, Gullu YT, Temocin F, Terzi O (2022). Clinical evaluation of patients with COVID-19 within the framework of comorbidities. Sisli Etfal Hastan Tip Bul.

[6] Godino C, Scotti A, Maugeri N, Mancini N, Fominskiy E, Margonato A, Landoni G (2021). Antithrombotic therapy in patients with COVID-19? -Rationale and evidence. Int J Cardiol.

[7] Emekli E, Yıldırım M, Özlü Ç (2022). Superior mesenteric artery thrombosis as a complication of COVID-19 pneumonia: a case report. Gazi Med J.

[8] Hajian A (2022). A case series of life-threatening hemorrhagic events in patients with COVID-19. Indian J Surg.

[9] Moores LK, Tritschler T, Brosnahan S (2020). Prevention, diagnosis, and treatment of VTE in patients with coronavirus disease 2019: CHEST guideline and expert panel report. Chest.

[10] Miesbach W, Makris M (2020). COVID-19: coagulopathy, risk of thrombosis, and the rationale for anticoagulation. Clin Appl Thromb Hemost.

[11] Nematihonar B, Qaderi S, Shah J, Bagherpour JZ (2021). Spontaneous giant rectus sheath hematoma in patients with COVID-19: two case reports and literature review. Int J Emerg Med.

[12] (2022). World Health Organization: clinical management of COVID- 19: interim guidance. https://apps.who.int/iris/handle/10665/332196.

[13] Turkish Ministry of Health (2022). Turkish Ministry of Health: COVID-19 (SARS-CoV-2 enfeksiyonu) antisitokin-antiinflamatuar tedaviler, koagülopati yönetimi. https://covid19.saglik.gov.tr/TR-66299/covid-19-tedavi.html.

[14] Angeramo CA, Méndez P, Eyheremendy EP, Schlottmann F (2022). Rectus sheath hematoma: conservative, endovascular or surgical treatment? A single-center artificial neural network analysis. Eur J Trauma Emerg Surg.

[15] Osinbowale O, Bartholomew JR (2008). Rectus sheath hematoma. Vasc Med.

[16] Chan NC, Weitz JI (2020). COVID-19 coagulopathy, thrombosis, and bleeding. Blood.

[17] Alakuş Ü, Kara U, Çimen Ş, Taşçı C, Eryılmaz M (2022). Life-threatening hematomas in COVID-19 patients. Ulus Travma Acil Cerrahi Derg.

[18] Özer M, Terzioglu SG, Keskinkılıç Yağız B, Gürer A, Dinç T, Coskun A (2022). Does COVID-19 increase the incidence of spontaneous rectus sheath hematoma?. Ulus Travma Acil Cerrahi Derg.

[19] Cherry WB, Mueller PS (2006). Rectus sheath hematoma: review of 126 cases at a single institution. Medicine (Baltimore).

[20] Hatjipetrou A, Anyfantakis D, Kastanakis M (2015). Rectus sheath hematoma: a review of the literature. Int J Surg.

[21] Yamada Y, Ogawa K, Shiomi E, Hayashi T (2010). Images in cardiovascular medicine. Bilateral rectus sheath hematoma developing during anticoagulant therapy. Circulation.

[22] Kaya C, Idiz UO, Yazıcı P, Bozkurt E, Ömeroğlu S, Ünlü MT, Mihmanlı M (2017). Is conservative management of spontaneous rectus sheath hematoma effective?. Med Bull Sisli Etfal Hosp.

[23] Tomizawa M, Shinozaki F, Hasegawa R (2017). Abdominal ultrasonography for patients with abdominal pain as a first-line diagnostic imaging modality. Exp Ther Med.

[24] Cocco G, Ricci V, Boccatonda A, Stellin L, De Filippis G, Soresi M, Schiavone C (2021). Sonographic demonstration of a spontaneous rectus sheath hematoma following a sneeze: a case report and review of the literature. J Ultrasound.

[25] Ruff AN, Cornelson SM, Panter AS, Kettner NW (2020). Rectus abdominis muscle tear diagnosed with sonography and its conservative management. J Ultrasound.

[26] Draghi F, Zacchino M, Canepari M, Nucci P, Alessandrino F (2013). Muscle injuries: ultrasound evaluation in the acute phase. J Ultrasound.

[27] Lehto M, Alanen A (1987). Healing of a muscle trauma. Correlation of sonographical and histological findings in an experimental study in rats. J Ultrasound Med.

[28] Pierro A, Cilla S, Modugno P, Centritto EM, De Filippo CM, Sallustio G (2018). Spontaneous rectus sheath hematoma: the utility of CT angiography. Radiol Case Rep.

[29] Berná JD, Zuazu I, Madrigal M, García-Medina V, Fernández C, Guirado F (2000). Conservative treatment of large rectus sheath hematoma in patients undergoing anticoagulant therapy. Abdom Imaging.

[30] Zissin R, Gayer G, Kots E, Ellis M, Bartal G, Griton I (2007). Transcatheter arterial embolisation in anticoagulant-related haematoma--a current therapeutic option: a report of four patients and review of the literature. Int J Clin Pract.

